# Rationale and Design of JenaMACS—Acute Hemodynamic Impact of Ventricular Unloading Using the Impella CP Assist Device in Patients with Cardiogenic Shock

**DOI:** 10.3390/jcm11154623

**Published:** 2022-08-08

**Authors:** Franz Haertel, Karsten Lenk, Michael Fritzenwanger, Ruediger Pfeifer, Marcus Franz, Nedim Memisevic, Sylvia Otto, Bernward Lauer, Oliver Weingärtner, Daniel Kretzschmar, Gudrun Dannberg, Julian Westphal, Laura Baez, Jurgen Bogoviku, P. Christian Schulze, Sven Moebius-Winkler

**Affiliations:** 1 Department of Cardiology, University Hospital Jena, Am Klinikum 1, 07747 Jena, Germany; 2 Department of Cardiology, University Hospital Leipzig, Liebigstrasse 20, Haus 4, 04103 Leipzig, Germany

**Keywords:** Impella, acute heart failure, intensive care, shock, extracorporeal circulatory support

## Abstract

Introduction: Cardiogenic shock due to myocardial infarction or heart failure entails a reduction in end organ perfusion. Patients who cannot be stabilized with inotropes and who experience increasing circulatory failure are in need of an extracorporeal mechanical support system. Today, small, percutaneously implantable cardiac assist devices are available and might be a solution to reduce mortality and complications. A temporary, ventricular, continuous flow propeller pump using magnetic levitation (Impella^®^) has been approved for that purpose. Methods and Study Design: JenaMACS (Jena Mechanical Assist Circulatory Support) is a monocenter, proof-of-concept study to determine whether treatment with an Impella CP^®^ leads to improvement of hemodynamic parameters in patients with cardiogenic shock requiring extracorporeal, hemodynamic support. The primary outcomes of JenaMACS are changes in hemodynamic parameters measured by pulmonary artery catheterization and changes in echocardiographic parameters of left and right heart function before and after Impella^®^ implantation at different support levels after 24 h of support. Secondary outcome measures are hemodynamic and echocardiographic changes over time as well as clinical endpoints such as mortality or time to hemodynamic stabilization. Further, laboratory and clinical safety endpoints including severe bleeding, stroke, neurological outcome, peripheral ischemic complications and occurrence of sepsis will be assessed. JenaMACS addresses essential questions of extracorporeal, mechanical, cardiac support with an Impella CP^®^ device in patients with cardiogenic shock. Knowledge of the acute and subacute hemodynamic and echocardiographic effects may help to optimize therapy and improve the outcome in those patients. Conclusion: The JenaMACS study will address essential questions of extracorporeal, mechanical, cardiac support with an Impella CP^®^ assist device in patients with cardiogenic shock. Knowledge of the acute and subacute hemodynamic and echocardiographic effects may help to optimize therapy and may improve outcome in those patients. Ethics and Dissemination: The protocol was approved by the institutional review board and ethics committee of the University Hospital of Jena. Written informed consent will be obtained from all participants of the study. The results of this study will be published in a renowned international medical journal, irrespective of the outcomes of the study. Strengths and Limitations: JenaMACS is an innovative approach to characterize the effect of additional left ventricular mechanical unloading during cardiogenic shock via a minimally invasive cardiac assist system (Impella CP^®^) 24 h after onset and will provide valuable data for acute interventional strategies or future prospective trials. However, JenaMACS, due to its proof-of-concept design, is limited by its single center protocol, with a small sample size and without a comparison group.

## 1. Introduction

Cardiogenic shock is characterized as a life-threatening, clinical syndrome that may present as an acute illness or an acute exacerbation of a chronic process. Acute myocardial infarction is the most common cause of this primary cardiac disorder and main cause of death [1,2]. Affected patients are critically ill and show inadequately low cardiac output that leads to circulatory failure with clinical and biochemical evidence of end-organ hypoperfusion, tissue hypoxia and consecutive or simultaneous malfunctions of vital organs [3].

The fundamental pathophysiological aspect of cardiogenic shock is a profound depression of myocardial contractility, ineffective cardiac stroke volume and failed circulatory compensation that perpetuate a vicious, downward spiral of reduced cardiac output, low blood pressure, low coronary perfusion and, eventually, further impairment of contractility [3].

A normal heart has a trapezoidal ventricular pressure-volume loop depending on ventricular preload and afterload [3]. Cardiogenic shock shows a restrictive patter with increased preload, afterload, end-diastolic pressure and low stroke volume leading to an increase in myocardial oxygen consumption [4]. Compensatory physiologic mechanisms allow transient stabilization of hemodynamics, but these mechanisms become pathological over time and lead to shock progression [3].

Clinically, this presents as hypotension refractory to volume resuscitation requiring further therapy intensification [5]. Multiorgan failure is common and long-term survival shows poor outcomes [6].

About 140,000 patients develop myocardial infarction every year in Germany [7]. Of these, approx. 10,000 patients (5–8%) develop cardiogenic shock [8]. Mortality of patients with cardiogenic shock due to myocardial infarction or heart failure remains very high with rates of 30–70% [8,9,10,11,12,13]. While recommended in current guidelines as a means for short term mechanical circulatory support (MCS, level of recommendation IIa, level of evidence C), there is a lack of evidence from randomized trials for cardiac assist devices in cardiogenic shock [14]. Available devices impact the circulatory system in distinct ways affecting preload, afterload, and contractility [15].

Aggressive treatment with inotropes and vasopressors has the potential to exacerbate the downward spiral of shock and results in higher myocardial oxygen consumption, increased afterload and impaired microcirculation [16].

The largest randomized trial of MCS in patients with CS, IABP Shock II, did not show any advantage of mechanical cardiac support using intra-aortic balloon counter-pulsation (decrease in afterload without active unloading of the failing left ventricle) compared to a conservative medical strategy of shock treatment [17]. Implantation of mechanical assist devices such as veno-arterial extracorporeal membrane oxygenation (VA-ECMO) is currently the only therapeutic option for stabilizing refractory cardiac shock patients [17,18,19]. However, due to the invasiveness of the procedure, there is a risk of bleeding, stroke, infections, sepsis, and limb ischemia [1]. In addition, VA-ECMO increases left ventricular end-diastolic pressure as well as afterload that again leads to increased wall stress and myocardial oxygen demand [20].

In contrast, Impella^®^ is a mechanical unloading device through placement in the left ventricle with continuous blood flow from the left ventricular cavity to the ascending aorta. It has been approved for the stabilization of patients with cardiogenic shock. It facilitates unloading of the left ventricle to support contractility independently from heart function and rhythm via adjustable flow rates. A dissociation of left ventricular peak pressure from aortic pressure can be achieved with higher mean arterial pressure. The pressure-volume loop of the left ventricle is shifted leftwards resulting in a lower left ventricular end diastolic pressure, a diminished pressure-volume area, lower left atrial and pulmonary wedge pressure, decreased myocardial oxygen consumption, and a reduction in the left ventricular wall tension [4]. Nevertheless, no study has systematically analyzed markers of myocardial unloading using Impella^®^ in patients with cardiogenic shock.

## 2. Methods and Analysis

### 2.1. Study Design

**Figure 1:**
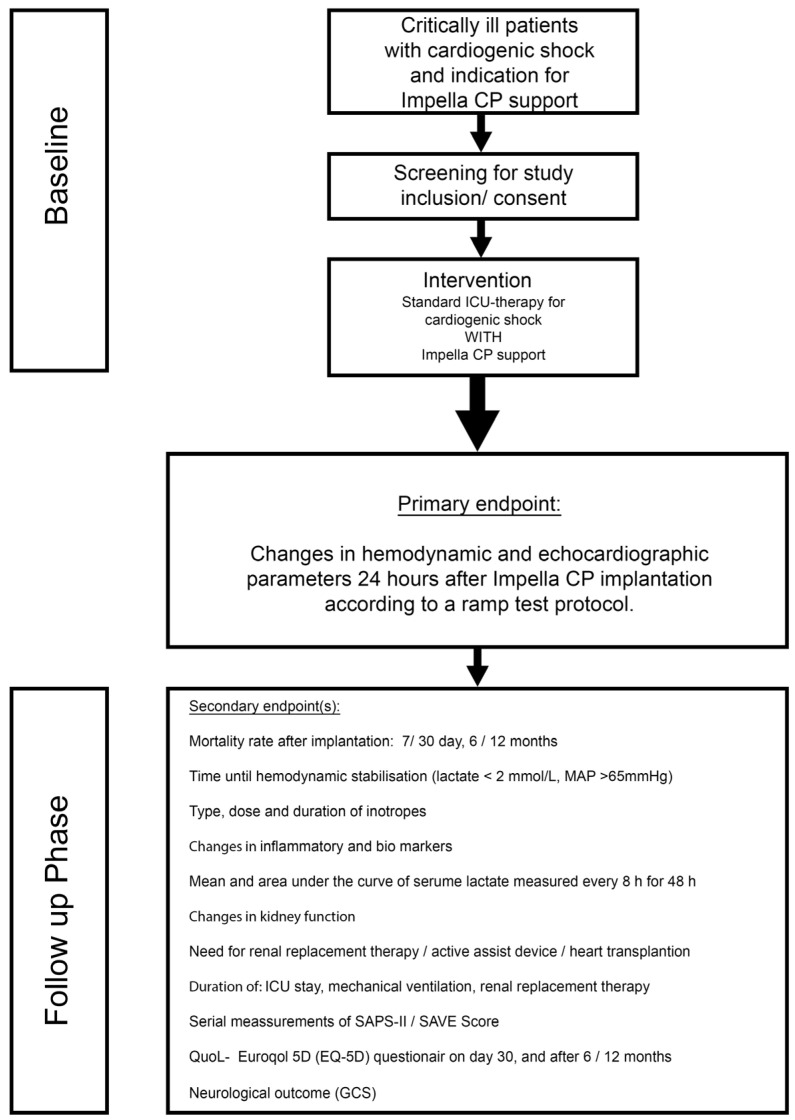
Flow chart of the study design. PAC = Pulmonary artery catheter; MAP = Mean arterial pressure; SAPS = Simplified acute physiology score; SAVE = Survival after VA ECMO; ICU = Intensive care unit; GCS = Glasgow outcome scale.

JenaMACS (Jena Mechanical Assist Circulatory Support) is a monocenter, prospective, open-labeled, proof-of-concept register study. JenaMACS will assess the impact of the Impella CP^®^ assist device on acute and subacute hemodynamic effects after implantation in patients with cardiogenic shock in addition to standard medical therapy. Figure 1 shows the study flow.

### 2.2. Patient and Public Involvement

Patients or the public were not involved in the design, or conduct, or reporting, or dissemination plans of our research.

### 2.3. Study Population

JenaMACS includes critically ill patients with cardiogenic shock and indication for an extracorporeal, mechanical, circulatory assist device determined by corresponding PAC (pulmonary artery catheter) hemodynamics (mean arterial pressure (MAP), pulmonary wedge pressure (PCPW), pulmonary artery pressure (PAP), pulmonary vascular resistance (PVR), systemic vascular resistance (SVR), mixed venous oxygen saturation (mvO2Sat), SV (stroke volume)). Inclusion and exclusion criteria are listed in Table 1.

### 2.4. Screening

All patients referred to our department with cardiac shock requiring hemodynamic support via an MCS device and that are eligible for Impella CP^®^ implantation will be screened.

### 2.5. Obtaining Informed Consent

The nature, aim and full extent of study participation are explained to each subject (or legally authorized representative or independent medical doctor) before inclusion. Informed consent will be obtained according to the Declaration of Helsinki.

There are two main modes of obtaining informed consent according to the different presentations of the patients: (1) Patients that are awake and have no cognitive impairment due to the cardiogenic shock will sign the informed consent prior to study initiation. A short and long version of the informed consent is available. (2) Patients with cognitive impairment due to the cardiogenic shock or that are sedated: two independent physicians will proof the presumed will of the patient. The patient will sign the consent form once he/she is recovered, or may not accept to be enrolled in the study and have to be excluded. Patients have the right to withdraw from study participation at any time without an explanation.

### 2.6. Objectives

#### 2.6.1. Primary Objective

The primary objective of the JenaMACS register is to investigate the potential benefit of the Impella CP^®^ on hemodynamics and left ventricular unloading in patients with cardiogenic shock after 24 h by using a ramp test protocol (Table 2 and Figure 1). The ramp test is performed as follows: During a step by step reduction of the Impella^®^ flow support (ramp test), hemodynamic (PCWP, SV) and echocardiographic parameters (left ventricular ejection fraction (LVEF), left ventricular end-diastolic diameter (LVEDD), and grading of mitral valve regurgitation) are recorded at each level from highest (P8) to lowest (P2). Measurements take place 10 min after adjusting the console to each flow level. In theory, mechanical assistance for unloading of the left ventricle might lead to a faster decrease in LVEDD, PCWP and mitral regurgitation and faster increase in LVEF and SV that could be translated into improved left ventricular recovery

#### 2.6.2. Secondary Objective

The secondary objectives are to gain information via serial hemodynamic measurements during Impella CP^®^ therapy in the time course, early from the start (10 min) to beyond 24 h, until explanation. Additionally, serial echocardiography will help to detect the effect of the Impella^®^ support to various echocardiographic parameters such as left heart size and right ventricular function. Apart from laboratory parameters, the secondary objectives mostly include clinical surrogate measures (e.g., mortality, time to lactate normalization intensive care unit (ICU) stay or time of mechanical ventilation) to identify or confirm potential advantages of Impella CP^®^ during the therapy and follow-up phase. Moreover, safety data like major and minor bleedings, stroke, sepsis and acute limb ischemia requiring intervention or surgery will be evaluated. Data acquisition during hospitalization and follow-up is displayed in Table 3.

### 2.7. Endpoints

**Figure 2:**
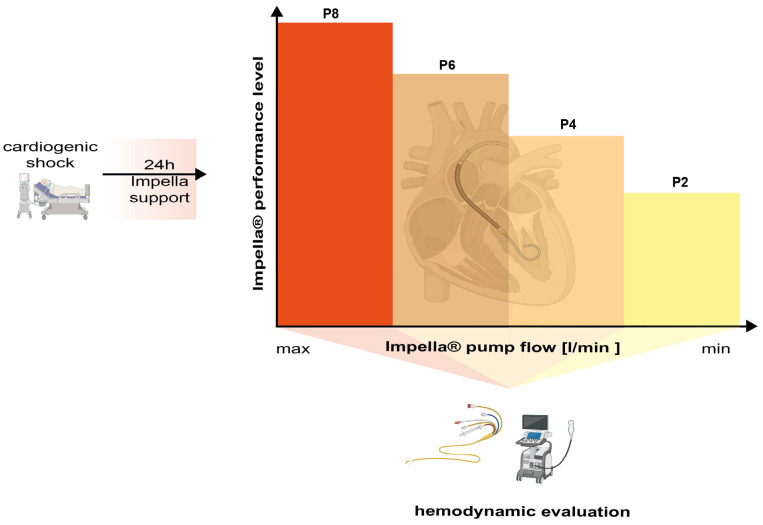
Study set up for measuring the primary outcome after 24 h of Impella^®^ support post-implantation via PAC and TTE(Graphic generated using BioRender©).

The primary endpoint of the JenaMACS register study is the change in hemodynamic and echocardiographic parameters after 24 h during the ramp test at different Impella^®^ support levels. Parameters to be measured are listed in Table 2 and a scheme is provided in Figure 2. Secondary endpoints are listed in Table 4.

### 2.8. Time Schedule, Study Duration and Frequency of Study Visits

The study duration is planned for 60 months: 57 months of recruitment, 3 months for final follow-up and another 3 months for data analysis and publication. Table 3 outlines the study visits. The primary endpoint is measured after 24 h by using a ramp test (Table 2 and Figure 2). Follow-up of the patients is 12 months in total after the beginning of the Impella CP^®^ implantation.

### 2.9. Technology to Be Used

#### 2.9.1. Impella^®^

The Impella CP^®^ (Abiomed, Danvers, MA, USA) used for JenaMACS is an axial, non-pulsatile, continuous 14 Fr magnetic levitation propeller pump delivering a maximum flow of 3–4 L per minute. Inserted percutaneously via Seldinger technique, the Impella^®^ is then advanced retrograde from the common femoral artery, through the aorta and across the aortic valve into the LV. An ultrasound-assisted puncture of the femoral artery can be performed to minimize access site complications. The device is using an impeller to eject blood from the left ventricle into the ascending aorta via a tube. The pump is not dependent on pulsatile flow or EKG. The impeller speed defines the flow rate of the device and therefore the blood volume to be ejected. Maximal flow rate for ImpellaCP^®^ is 3.5 L/min. Flow rate adjustments can be set manually from an external console and are the basis of the ramp test 24 h after Impella CP^®^ implantation (Table 2 and Figure 2). Flow rate also depends on the preloading of the left ventricle.

#### 2.9.2. Pulmonary Artery Catheter (Swan-Ganz—Catheter)

To quantify the extend and severity of cardiac shock and improvement under mechanical cardiac support (MCS), hemodynamic measurements will be obtained by pulmonary artery catheter and include pulmonary capillary wedge pressure (PCWP), mean right atrial pressure (RA) central venous oxygen saturation (cvSo2), and mixed venous oxygen saturation (mvSO2).

The following parameters will be calculated: cardiac index (CI), stroke volume (SV), systemic vascular resistance (SVR), pulmonary vascular resistance (PVR), and cardiac output (CO). At the trial site, the pulmonary artery catheter Corodyn TD^®^ (B. Braun^®^, Melsungen, Germany) will be used. This 4-lumen catheter is inserted with the cardiac catheterization lab either through the femoral or jugular vein before Impella^®^ placement and has a caliber of 7 Fr and a length of 110 cm.

#### 2.9.3. Transthoracic Echocardiography

Transthoracic echocardiography (TTE) is performed using the ultrasound system Vivid S70^®^ (GE^®^, Boston, MA, USA) and the recordings are processed via dedicated software (Image-Arena ™ Version 4.6; TomTec Imaging Systems, Unterschleissheim, Germany). Echocardiographic evaluation is carried out in a standardized comprehensive fashion and encompasses acquisition of features of left/right ventricular and valvular function (Table 2).

### 2.10. Safety, Possible Complications and/or Risks

Study participation is not associated with any additional risks for patient’s safety. The Impella CP^®^ is a CE-approved medical device and part of routine, clinical use for cardiogenic shock and other indications. The use of a Swan-Ganz Catheter is standard practice in cardiogenic shock patients. For safety assessments, documentation of relevant events regarding Impella CP^®^ treatment include the aspects listed in Table 5.

### 2.11. Statistical Considerations and Methods

#### 2.11.1. Sample Size

The planning aims to obtain data on hemodynamic parameters and differences in hemodynamics in the clinical course of 20 patients.

#### 2.11.2. Statistical Analyses

The analysis of the primary endpoint and the analysis of the secondary endpoints are carried out on the basis of the protocol. Sensitivity analysis is carried out on the basis of the Per-Protocol-Set (PPS). The analysis includes all patients, who were included, whose declaration of consent is available and who received the planned therapy. The PPS analysis population includes all patients of the ITT who did not have a serious protocol violation. For secondary analysis, all patients are included who have a complete data set for the endpoint under consideration.

The primary analysis will be performed on the full analysis set which includes all patients treated. The secondary endpoint analysis is carried out descriptively using statistical test procedures. The evaluation of frequencies is done by Chi-square test/Fisher’s exact test. The evaluation of metric endpoints is carried out using an analysis of variance. Time-to-event endpoints are evaluated using Kaplan-Meier estimates and a log-rank test. In addition to the test results, effect estimates with 95% confidence intervals are given. Odds ratio will be calculated as an estimator of the effect of MCS treatment. To investigate multivariable relationships, multiple logistic regression (binary endpoints, including the primary endpoint), linear model (for quantitative endpoints) or Cox regression (long-term survival) are carried out. In addition, the logistic regression model will be used to evaluate the prognostic value of parameters of hemodynamics and to differentiate which might be confounders. Lists and tabular summaries are created to describe the adverse and serious adverse events. Predefined subgroup analysis is carried out for gender, age groups > 75 years, diabetes, arterial hypertension, ST-elevation myocardial infarction (STEMI) versus Non ST-elevation myocardial infarction (NSTEMI), anterior wall infarction versus infarction in other locations, previous infarction. According to the data distribution, two-sided independent samples t test or Mann-Whitney-U test will be applied for continuous endpoints. Safety analyses will be run in the safety population to be defined in the protocol; these analyses will summarize and tabulate all observed safety events including a measure of uncertainty (i.e., 95% confidence intervals). The confirmatory analysis will be performed at a significance level of 5%.

### 2.12. Study Inclusion and Patient Treatment

Patients with cardiogenic shock are screened and transferred to the cardiac catheterization laboratory as soon as possible after admission or after being transferred from another hospital. After checking the inclusion/exclusion criteria and receiving consent, the patient will be included in the study. Left heart and right heart catheter examinations are performed and the coronary vessel responsible for the infarct is recanalized using percutaneous coronary intervention (PCI) as soon as possible. In case of an indication for immediate bypass surgery, the study is not discontinued, but further evaluation according to the intention-to-treat principle will apply. Further medicinal measures for the treatment of acute myocardial infarction, circulatory-support therapy with catecholamines, assisted or volume-controlled ventilation, etc., are carried out according to the decision of the physician in charge according to recognized therapy guidelines.

#### 2.12.1. Documentation of Hemodynamic Parameters

Hemodynamic parameters such as arterial systolic and diastolic blood pressure, mean blood pressure and heart rate are documented in the cardiac catheterization laboratory prior to the implantation of Impella CP^®^. Pulmonary artery catheter examination will measure the various pressures (e.g., diastolic, systolic and mean pulmonary artery pressure (PAP), pulmonary capillary wedge pressure (PCWP)) and resistances (e.g., systemic and pulmonary vascular resistance). This is repeated at defined intervals together with an arterial and mixed venous blood gas analysis for determination of pH, serum lactate and standard base excess.

#### 2.12.2. Echocardiography

Echocardiography is performed before and after Impella CP^®^ implantation as well as on a daily basis until the Impella CP^®^ is explanted. Parameters of the left and right ventricular size and function as well as the documentation of possible valve abnormalities are standard and will be stored within the internal Picture Archiving and Communication System (PACS). Parameters are listed in Table 2.

#### 2.12.3. Additional Documentation of Hemodynamic Parameters

At the end of the examination in the catheterization laboratory, the hemodynamic parameters from pulmonary artery catheterization are documented again about 30 min after implantation of the Impella CP^®^. Furthermore, a new arterial and mixed venous blood gas analysis is performed with additional determination of pH, serum lactate and standard base excess. Echocardiographic parameters are recorded according to the protocol.

#### 2.12.4. Post-Implantation

The patients are monitored until they are transferred to the intensive care unit. The flow of the device should be optimized according to clinical goals (MAP ≥ 65 mmHg).

#### 2.12.5. Supportive Therapy in the Intensive Care Unit

Further intensive medical therapy is carried out according to generally recognized guidelines. Systemic anticoagulation with unfractionated heparin will be used for the duration of left ventricular support (target activated clotting time around 180 s).

Drug therapy with catecholamines (noradrenaline, dobutamine, possibly suprarenin, vasopressin) according to current recommendations is possible. The use of calcium sensitizers (e.g., levosimendan) or other hemodynamically relevant drugs (e.g., PDE inhibitors) are also possible according to an individual intensive care medicine assessment [14]. The aim, independent of the study, is to achieve a mean arterial blood pressure of at least 65 mmHg. All patients receive an invasive hemodynamic measurement using a standard pulmonary artery catheter. The daily doses of catecholamines and other hemodynamic drugs are documented. Furthermore, the daily fluid balance/management up to the explanation are recorded. If the use of a renal replacement therapy is necessary, the duration and type of the procedure are documented. In addition, the duration of any mechanical ventilation, the time of extubation and any reintubation that may be necessary are documented. Left ventricular ejection fraction is determined again within 24 h and after 7 days.

As part of the recording of the intensive medical treatment, the SAPS (Simplified acute physiology score) II score is recorded serially every day in the intensive care unit. To determine inflammatory parameters, as well as liver and kidney functions, a full blood work-up is recorded daily including C-reactive protein (CRP) and procalcitonin (PCT). All other laboratory parameters are determined at least once a day according to the documentation sheets in the routine laboratory program. Ethylenediaminetetraacetic acid (EDTA), blood, serum and plasma are stored at all times (before, after implantation, day 1–3 and 7) for later molecular biological analysis.

#### 2.12.6. Hemodynamic Monitoring in the Intensive Care Unit

At least three times a day the measuring of selected hemodynamic parameters takes place and includes: arterial systolic/diastolic blood pressure, mean arterial pressure, heart, arterial blood gas analysis with additional determination of pH, base excess, serum lactate and parameters from invasive monitoring via PAC (pulmonary capillary wedge pressure (PCPW), pulmonary artery pressure (PAP), cardiac output (CO), central venous pressure (CVP), mixed venous oxygen saturation (mvO_2_Sat), pulmonary vascular resistance (PVR), and systemic vascular resistance (SVR)).

#### 2.12.7. Weaning and Explantation of the Impella CP^®^ Device

The duration of therapy is determined by the need for supportive therapy with catecholamines. In previous studies, the mean duration of mechanical circulatory support was 3–4 days [21]. The main criterion for starting weaning from mechanical circulatory support is sustained hemodynamic stability (systolic RR > 90 mmHg for >60 min) without therapy with catecholamines and without signs of peripheral end-organ hypoperfusion. Impella^®^ flow is reduced in steps every 3 h; after 3 h at level 2, another echocardiography, hemodynamic measurement and blood sampling followed by explanation of the Impella^®^ take place. Standardized femoral artery access site closure takes place after explantation via a suture mediated percutaneous system as a first option (Perclose ProGlide^®^, Abbott laboratories, Abbott Park, Chicago, IL, USA)

#### 2.12.8. 30 Day Follow-Up

A new echocardiographic assessment is carried out before discharge from the hospital. After 30 days, the patient’s vital status is again ascertained through contact. All clinical events are verified by obtaining the original documentation. In addition, dyspnea according to the New York Heart Association (NYHA)-classification, angina pectoris according to the Canadian Cardiovascular Society (CCS)-classification and quality of life according to the EuroQol (EQ)-5D questionnaire are recorded.

#### 2.12.9. Follow-Up Plan

Further treatment of the patients after the end of the clinical trial is carried out by the general practitioners/resident and/or cardiologists. In the event of undesirable side effects, patients are instructed to contact the study director or one of his representatives in accordance with Section 7, Paragraph 2, Item 13 of the Good Clinical Practice (GCP)-ordinance.

### 2.13. Methods against Bias

#### 2.13.1. Blinding and Treatment Bias

Blinding is not possible because of the intervention used. Methods used against bias are standardized examination protocols to even out systematic differences due to differing expertise at all levels, and an independent clinical endpoint committee to evaluate adverse events other than death; high clinical standards are maintained with regard to the number of PCIs performed/year (about 1000) of patients treated in cardiogenic shock and participation in clinical studies. Furthermore, our study center has a certified, cardiac ICU specialized in the treatment of cardiac shock and application of Impella CP^®^ that are embedded in a quality management system. Investigators are trained in good clinical practice (GCP). All medical professionals involved are qualified and hold the appropriate national diploma or at least operate under surveillance of a senior staff member.

#### 2.13.2. Measurement Bias

At each visit, the measured parameters are documented in the case report forms (CRFs). In addition, all patients will be followed-up after screening and implantation of the Impella CP^®^ system. Finally, monitoring (on-site and central) will be carried out during the trial by staff of the Department of Cardiology and of the Center for Clinical Studies, Jena University Hospital.

### 2.14. Treatment Compliance/Drop-Out Rate at Follow-Up

Treatment compliance is not influenced by the patient as the patient is normally unconscious and in a critical clinical condition. As a result, 100% treatment compliance is assumed. Loss of follow-up is estimated to be less than (<4%) based on other clinical studies in cardiogenic shock [9,11,12].

### 2.15. Data Management

#### 2.15.1. Data Assessment/Case Report Forms

Paper CRF and a web application for study management software are used for data documentation (Redcap Database). The data will be stored online in REDCap (Research Electronic Data Capture, REDCap Consortium, Vanderbilt University (USA)). REDCap is a secure web application for building and managing online surveys and databases. The software meets the regulatory requirements (GCP, 21CFR Part11). The data will be entered via encrypted connection (HTTPS) in web browser input masks. Each subject will be given an unambiguous patient identification number to ensure pseudonymized data analysis. The data are verified by range, validity and consistency checks. The data manager will demand clarification of missing or implausible data from the study teams. Each change in data, e.g., due to resolved queries, will be documented in the data base by an automated audit trail. Unauthorized data access is prohibited by a hierarchical concept of access based on users and roles.

#### 2.15.2. Ethics Approval

The study was approved by the institutional review board and ethics committee of the Friedrich Schiller University (registration number: 4939-10/16).

#### 2.15.3. Privacy, Collection and Processing of Data

All data/information obtained during the study are subject to appropriate use and protection and will be treated in accordance with the comprehensive regulations of the national data protection law in strictest confidence. During the study, subjects will be identified exclusively by an individual identification code (subject number). For protection of these data, organizational procedures are implemented to prevent distribution of data to unauthorized parties. The appropriate regulations of local data legislation will be fulfilled in their entirety.

#### 2.15.4. Dissemination

All findings of this study will be published in a renowned international medical journal, regardless of the outcomes. We used the SPIRIT reporting guidelines for reporting [22].

## 3. Summary and Conclusions

Although MCS-treatment with Impella CP^®^ is an option in cardiogenic shock, data published assessing the use of Impella^®^ and its impact are too small to draw definite conclusions with regard to clinical endpoints such as unloading characteristics, hemodynamics or mortality. The only currently recruiting larger randomized clinical trial powered for clinical endpoints—DanGer-Shock—will still need several additional years until finalization [23]. The implementation of the Impella CP^®^ in patients with cardiogenic shock offers rapid improvement from circulatory failure and increases in end-organ tissue perfusion in a less invasive rescue strategy. This could be the optimal device for patients with cardiogenic shock, reducing the rate of severe bleeding events, systemic inflammatory response syndrome, or limb ischemic events. Furthermore, left ventricular performance might also be positively influenced as a result of direct cardiac unloading an antegrade, more physiological outflow into the acceding aorta. However, the Impella CP^®^ has a flow limitation of maximally 3.5 L/min (under laboratory model conditions using water) and is limited to compensation for left ventricular failure. By contrast, this technology is not able to support profound, critical states of shock with coexisting condition that need combined respiratory and (biventricular) circulatory therapy. Prospective clinical studies such as the JenaMACS trial are therefore warranted.

## Figures and Tables

**Table 1:** Inclusion and exclusion criteria of the JenaMACS study.

Inclusion	Exclusion
- Systolic blood pressure < 90 mmHg, >30 min of inotropic agents and/or vasopressors to keep the blood pressure > 90 mmHg systolic - Signs of left ventricular failure with pulmonary congestion. - Signs of end-organ hypoperfusion with at least one of the following criteria: - Somnolence. - Cold, pale skin or extremities. - Oliguria (≤30 mL/h). - Serum lactate > 2.0 mmol/L. - Age ≥ 18 years ≤90 years. - Signed informed consent	- Contraindications to Impella CP^®^ implantation. - Shock duration > 12 h before evaluation. - Severe PVD making Impella implantation impossible. - Aortic valve insufficiency/stenosis at least II°. - Age > 90 years. - CNS disease with fixed, dilated pupils (not drug-induced). - Severe concomitant disease with limited life expectancy < 6 months. - Participation in another study. - CPR > 30 min. - Shock due to other reasons.

CNS = Central nervous system, CPR = Cardiopulmonary resuscitation, PVD = Peripheral vessel disease.

**Table 2:** Ramp Test protocol: A ramp test will be performed 24 h after Impella CP^®^ implantation according to the following protocol.

Speed	Flow	Mean BP	HR	PCWP	Mean PA	PAO2	Lactate	CI	MR	LVEDD	LVESD	LVEF	RVEDD
	[L/min]	[mmHg]	[L/min]	[mmHg]	[mmHg]	[%]	[mmol/L]		[Degree]	[mm]	[mm]	[%]	[mm]
**P8**													
**P6**													
**P4**													
**P2**													

BP = Blood pressure; HR = heart rate; PCWP = Pulmonary, capillary wedge pressure; PA = Pulmonary artery; CI = Cardiac index; LVEDD = Left ventricular end-diastolic diameter, LVESD = Left ventricular end-systolic diameter; LVEF = Left ventricular ejection fraction; RVEDD= Right ventricular end-diastolic diameter; T = Impella flow level, MR = Mitral regurgitation.

**Table 3:** Frequency and scope of the JenaMACS study visits.

					Time Point					
Protocol Item	Pre PCI	Post-PCI	Day 1	Day 2	Day 3-X *	Stabilization ^+^	Day 7/E	30 Rays	6 M.	12 M.
Inclusion/exclusion criteria	X									
Informed consent	X									
12-lead surface ECG	X	X	X	X	X	X	X	X		
Laboratory evaluation ^∆^	X		X	X	X	X	X			
Medical history, Comorbidities ^∆^	X									
Physical examination ^∆^	X	X	X	X	X	X	X	X	X	X
BGA including lactate	X	X	X	X	X	X				
Creatinine clearance **	X		X	X	X					
Blood Samples (Measurement of biomarkers)	X	X	X	X	X	X	X	X	X	X
PAC—Hemodynamics	X	X	X	X	X	X				
Measurement of parameters of hemodynamics ^∆^	X	X	X	X	X	X				
TTE	X	X	X	X	X	X	X	X		
Dose of isotopes and vasopressors	X	X	X	X	X	X	X	X		
Impella CP^®^ data	X	X	X	X	X	X				
SAPS-II Score		X	X	X	X	X				
CCS category								X	X	X
NYHA category								X	X	X
Quality of Life (EQ-5D)							X	X	X	X
Measurement of the neurological outcome (GCS) ^∆^	X	X	X	X	X	X	X	X	X	X

^∆^ Data documentation from clinical routine (if available), * every day until hemodynamic stabilization; ** Clearance calculated according to Cockcroft-Gault formula; ^+^ after Impella explantation; SAPS = Simplified Acute Physiology Score; ECG = Electrocardiography; TTE = Transthoracic echocardiogram; PAC = Pulmonary artery catheterization; NYHA = New York Heart Association; CCS = Canadian Cardiovascular Society; GCS = Glasgow coma scale; PCI = Percutaneous, coronary intervention; BGA = Blood gas analyses. EQ—5D = EuroQol questionnaire.

**Table 4:** Secondary endpoints of the JenaMACS study.

Secondary Endpoints
- Change of hemodynamic and echocardiographic parameters after 10 min, 30 min and every 8 h of Impella support - Need, type, dose and duration of inotropic/vasopressor treatment. - Time until hemodynamic stabilization (lactate < 2 mmol/L; mean arterial pressure > 65 mmHg) after implantation. - Change in inflammatory parameters over time. - Mean and area under the curve of serum-lactate measured every 8 h for 48 h after implantation. - Mortality defined as mortality rate in the hospital or anywhere after discharge, within 12 months after implantation. - Changes in kidney function after implantation. - Need and duration of renal replacement therapy (CVVHD) and mechanical ventilation after implantation. - Length of stay at ICU after implantation. - Need for an active assist device or heart transplantation after implantation. - SAPS II Score at baseline and every 24 h during Impella CP^®^ support. - EQ-5D-questionnaire including EQ VAS after implantation. - Neurological outcome (GCS) after implantation. - Safety aspects such as major bleeding according to GUSTO/BARC definition, critical limb ischemia, sepsis, hemorrhagic or ischemic stroke

CVVHD = continuous veno-venous hemodialysis, ICU = intensive care unit, SPAS = Simplified acute physiology score, EQ—5D = EuroQol questionnaire, VAS = Visual analogue scale, GCS = Glasgow Coma Scale BARC = Bleeding academic research consortium, GUSTO = Global use of streptokinase and t-PA for occluded coronary arteries, PCT = procalcitonin.

**Table 5:** Safety of the JenaMACS trial. Any of the mentioned adverse events (AE) is considered as a serious adverse event (SAE) if it is directly caused, may have caused in the past or may cause in the future death or a serious aggravation of the state of health of a patient, user or another person if the incident was caused by the medical device. The events will be recorded from the moment the medical device is unpacked and inserted into the patient until follow-up at discharge.

Safety of the Trial
- Peri-procedural complications within 7 days combined from: pericardial effusion, stroke, major bleeding, embolization, intracranial bleeding. - Device related: thrombus, breakage, malfunction, allergic reactions. - Vascular access related complications requiring intervention/surgery. - Procedure related death. - Post-procedure infection rate. - Sepsis with clinical signs of infection and increased PCT (>2 pg/mL). - Peri-procedural outcomes at 7 and 30 days after implantation. - Bleeding requiring transfusion (BARC 2—5). - Severe and moderate bleeding complications (GUSTO definition). - Stroke. - Severe limb ischemia/peripheral vascular complications.

BARC = Bleeding academic research consortium, GUSTO = Global use of streptokinase and t-PA for occluded coronary arteries, PCT = procalcitonin.

## Data Availability

The data presented in this study are available on request from the corresponding author. The data are not publicly available due to local legal restrictions on data safety.
